# Widely targeted metabolomics and machine learning identify succinate as a key metabolite in sepsis-associated encephalopathy

**DOI:** 10.1016/j.isci.2025.114520

**Published:** 2025-12-23

**Authors:** Hongjie Hu, Yikuan Feng, Yunxi Zhou, Shu Peng, Dayong Li, Shuhui Wu, Hebin Jiang, Yuru Lu, Jingbo Chen, Yaqin Song, Wei Zhu

**Affiliations:** 1 Department of Emergency Medicine, Tongji Hospital, Tongji Medical College, Huazhong University of Science and Technology, Wuhan, Hubei 430030, P.R. China; 2 Department of Intensive Care Medicine, Tongji Hospital, Tongji Medical College, Huazhong University of Science and Technology, Wuhan, Hubei 430030, P.R. China; 3 Department of Thoracic Surgery, Tongji Hospital, Tongji Medical College, Huazhong University of Science and Technology, Wuhan, Hubei 430030, P.R. China

**Keywords:** health sciences

## Abstract

Sepsis-associated encephalopathy (SAE) is a common and serious complication of sepsis that leads to acute brain dysfunction and long-term cognitive impairment. We used widely targeted LC-MS/MS plasma metabolomics in 29 healthy controls, 32 sepsis patients, and 27 SAE patients, combined with machine learning, to define metabolic patterns across these groups. This approach identified 12 discriminatory metabolites, with succinate showing a stepwise increase from health to sepsis to SAE and associations with clinical severity scores. To test its functional relevance, we used a cecal ligation and puncture (CLP) mouse model and found that exogenous succinate supplementation aggravated cognitive deficits, neuronal injury, and microglial activation. Together, these findings link systemic metabolic remodeling to brain inflammation and dysfunction in sepsis and suggest that succinate and related pathways may help stratify SAE risk and provide mechanistic entry points for future therapeutic exploration.

## Highlights

• Succinate increases from health to sepsis and SAE, serving as a key metabolic driver
• Machine learning-integrated metabolomics identifies robust biomarkers for SAE diagnosis
• Succinate supplementation aggravates cognitive deficits and neuroinflammation in CLP mice
• Dysregulated glycerophospholipid metabolism and fatty acid oxidation drive SAE progression

## Graphical abstract

**Figure:**
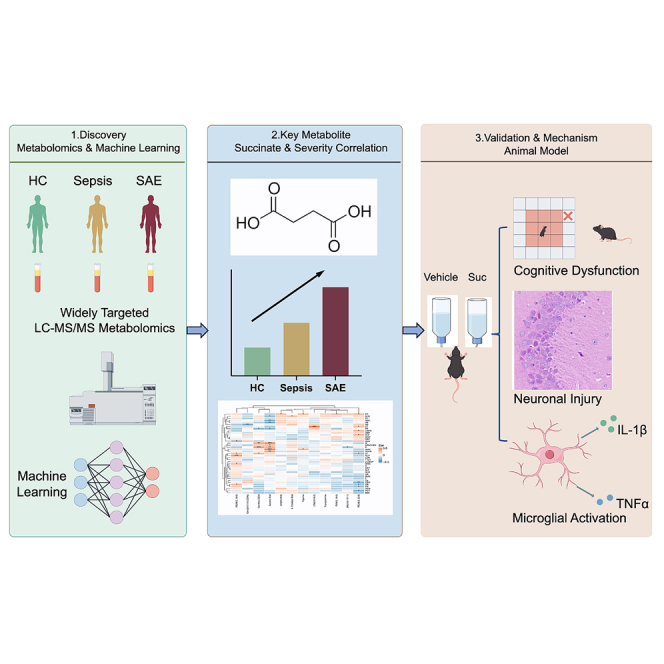

## Introduction

Sepsis-associated encephalopathy (SAE) is one of the most common organ dysfunctions in sepsis and a leading cause of encephalopathy in the intensive care unit (ICU). It is strongly associated with higher mortality, increased ICU resource utilization, and prolonged hospital stays.^1^ Clinically, SAE manifests as altered consciousness, cognitive impairment, and focal neurological symptoms.^2^^,^^3^ However, due to the non-specific nature of these clinical features,^4^ SAE is often overlooked or diagnosed late in clinical practice. Therefore, accurate and early assessment of cerebral function is crucial for the diagnosis, management, and prognosis of SAE.

Currently, the research on specific biomarkers for SAE faces challenges. Although reported biomarkers, such as neuron-specific enolase (NSE) and S100β are elevated in SAE patients, their insufficient sensitivity and specificity, coupled with their tendency to be abnormally elevated in various other neurological or systemic diseases.^5^^,^^6^ render them inadequate for clinical application. This bottleneck has led to a lack of reliable assessment tools, making it difficult to sensitively and specifically identify sepsis-associated brain dysfunction in clinical practice. Therefore, there is an urgent need to develop accurate methods for assessing SAE, elucidate its molecular mechanisms, and establish a multidimensional biomarker detection system.

Metabolites, as intermediates or end products of cellular metabolism, reflect the physiological state, energy metabolism, and organ function of the organism in a direct manner, serving as a “chemical barometer” of health.^7^ In sepsis, peripheral inflammatory cytokines activate immune responses in central nervous system glial cells (e.g., microglia and astrocytes),^8^ triggering metabolic reprogramming^9^ and the accumulation of abnormal metabolites,^10^ which may contribute to the development and occurrence of SAE. Thus, identifying highly specific metabolic biomarkers holds great value for early detection and diagnostic of SAE. However, clinical studies on metabolites related to SAE remain limited.

Metabolomics, as a systems biology-based approach, provides a comprehensive qualitative and quantitative assessment of endogenous metabolites. This methodology facilitates the identification of disease-associated metabolic signatures, enables pathway enrichment analyses, and reveals dynamic metabolic patterns. To balance coverage and detection performance, this study employed a widely-targeted metabolomics strategy to identify and quantify plasma metabolites. Combined with machine learning algorithms, we aimed to screen the most predictive metabolic biomarkers and establish an early diagnostic for SAE.

## Results

### Participants characteristics

A total of 88 participants were enrolled, including 29 HC, 32 patients with sepsis, and 27 patients with SAE. Baseline characteristics, including age, sex, and BMI, did not differ significantly among groups (all *p* > 0.05; Table 1). In contrast, laboratory findings demonstrated progressive abnormalities with increasing disease severity, reflected by elevated inflammatory markers, metabolic disturbances, and organ function indicators.

**Table 1:** Baseline demographic and clinical characteristics of participants in HC, Sepsis, and SAE groups

Characteristics	HC	sepsis	SAE	*P*
Number of patients	29	32	27	
Male (n, %)	16 (55)	18 (56)	10 (37)	0.218
Age	58 [50–66]	62 [54–71]	65 [49–70]	0.455
BMI (kg/m^2^)	22.6 [19.5–25.8]	21.8 [19.1–24.6]	22.5 [18.7–26.4]	0.421
Temperature (°C)	36.2 [36.1–36.6]	36.7 [36.4–37.9]	36.6 [36.4–38.3]	
Heart Rate (bpm)	71 [63–76]	105 [87–121]	101 [87–135]	<0.001
Respiratory rate	20.0 [19.3–23.1]	20.0 [18.8–25.5]	20.0 [19.5–25.5]	0.436
SBP (mmHg)	119.0 [110.0–131.0]	103.0 [78.0–114.0]	98.0 [86.0–120.0]	<0.001
DBP (mmHg)	75.0 [68.0–81.0]	58.0 [48.5–69.5]	65.0 [52.0–73.5]	<0.001
MBP (mmHg)	90 [82–99]	76 [59–94]	72 [54–90]	<0.001
APACHE II score		18 [11–25]	21 [14–29]	
SOFA score		8 [5–11]	10 [6–14]	
GCS Score		12 [10–15]	10 [8–11]	

**Laboratory findings**				

WBC (10^3^/mm^3^)	5.87 [4.73–6.88]	11.8 [7.8–17.7]	13.9 [9.3–22.2]	<0.001
RBC (10^6^/mm^3^)	4.64 [4.15–5.12]	3.75 [3.11–4.40]	3.49 [2.61–4.38]	<0.001
Hb(g/L)	140 [124–157]	112 [91–133]	104 [76–131]	<0.001
Platelet (10^3^/mm^3^)	213 [178–246]	161 [36–287]	132 [54–210]	<0.001
ALT (U/L)	15 [10–25]	19 [12–49]	26 [16–45]	0.013
AST (U/L)	21 [19–25]	33 [20–89]	43 [30–72]	<0.001
TP (g/L)	75.7 [70.7–80.8]	57.2 [48.9–65.5]	59.8 [50.1–69.3]	<0.001
Albumin (g/L)	45.0 [42.3–47.8]	29.5 [23.6–35.4]	30.6 [22.2–39.0]	<0.001
Globulin (g/L)	30.7 [27.7–33.6]	27.7 [21.8–33.5]	29.2 [22.5–35.8]	0.100
TBil (μmol/L)	15.3 [12.0–19.0]	12.7 [7.5–22.0]	17.4 [9.3–26.6]	0.614
DBil (μmol/L)	3.9 [3.2–5.8]	9.1 [3.6–15.3]	10.0 [4.6–15.4]	0.004
IBil (μmol/L)	5.0 [3.6–9.4]	4.6 [3.2–8.5]	5.7 [2.5–11.6]	<0.001
Cholesterol (mmol/L)	2.49 [2.06–3.24]	2.25 [2.03–2.98]	2.97 [2.26–3.63]	<0.001
Glucose (mmol/L)	7.8 [5.6–11.3]	7.7 [5.6–11.5]	7.9 [6.5–10.6]	<0.001
BUN (mmol/L)	8.8 [5.7–15.4]	7.1 [5.4–13.9]	9.6 [6.7–16.3]	<0.001
Creatinine (μmol/L)	130.8 [68.6–207.0]	98.0 [64.0–192.8]	141.9 [86.7–233.0]	0.003

APACHE II = acute physiology and chronic health evaluation II, SOFA = sequential organ failure assessment, GCS = Glasgow coma score.

### Integrated widely targeted metabolomics—machine learning workflow for discovery and stratification of SAE

**Figure 1:**
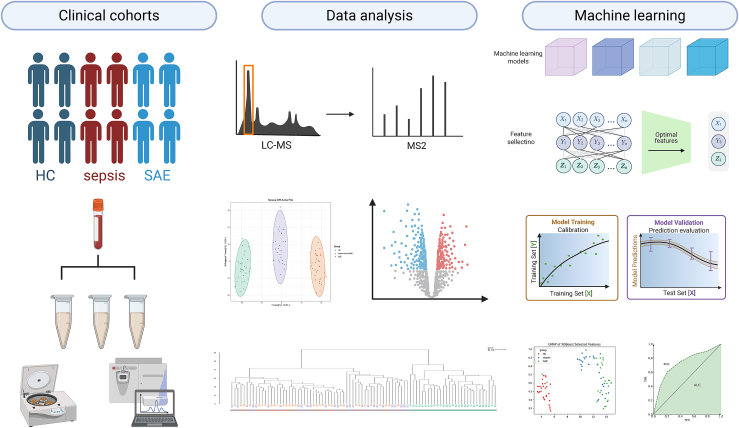
Integrated widely targeted metabolomics–machine learning workflow for discovery and stratification of SAE Plasma from HC, sepsis, and SAE patients underwent non-targeted LC-MS/MS profiling, yielding 1,307 high-confidence metabolic features. Multivariate analyses (OPLS-DA, UMAP) revealed clear group separation. Feature selection combined with classifier comparison identified optimal metabolite subsets. An XGBoost model was trained and calibrated, achieving robust SAE discrimination in an independent validation set (AUC = 0.92). Key metabolites contributing to classification were further interpreted for biological relevance.

To characterize plasma metabolic alterations across the SAE continuum and identify candidate biomarkers, we integrated clinical sampling, high-resolution widely targeted LC-MS/MS, multivariate feature selection, and supervised classification. As shown in Figure 1, peripheral blood plasma samples were obtained from three groups. HC were recruited from a health examination center. For septic patients, blood samples were collected within 24 h after the diagnosis of sepsis. The occurrence of SAE was subsequently determined during the clinical course, and patients who met the diagnostic criteria for SAE were classified into the SAE group for further analysis. The sample extracts were analyzed using an LC-ESI-MS/MS system (UPLC, ExionLC AD, https://sciex.com.cn/; MS, QTRAP6500+ System, https://sciex.com/).

Raw data were processed through peak picking, alignment, and deconvolution. After removal of background signals and redundant ions, 1,307 QC-vetted metabolite features were retained for downstream modeling and differential analysis. Unsupervised projection methods, including orthogonal partial least squares-discriminant analysis (OPLS-DA) and uniform manifold approximation and projection (UMAP), revealed clear separation among the HC, sepsis, and SAE groups in metabolic feature space.

To extract the most informative features, we applied recursive feature elimination combined with a multi-classifier comparison framework incorporating RF, LR, SVM, and XGBoost. Model performance was evaluated through cross-validation, followed by structural optimization, selection of the optimal feature subset, and probability calibration to improve reliability. The final XGBoost classifier demonstrated excellent generalizability on an independent test set, showing superior performance in distinguishing SAE from other phenotypes.

### Metabolomic profile alterations and biological pathways in SAE

**Figure 2:**
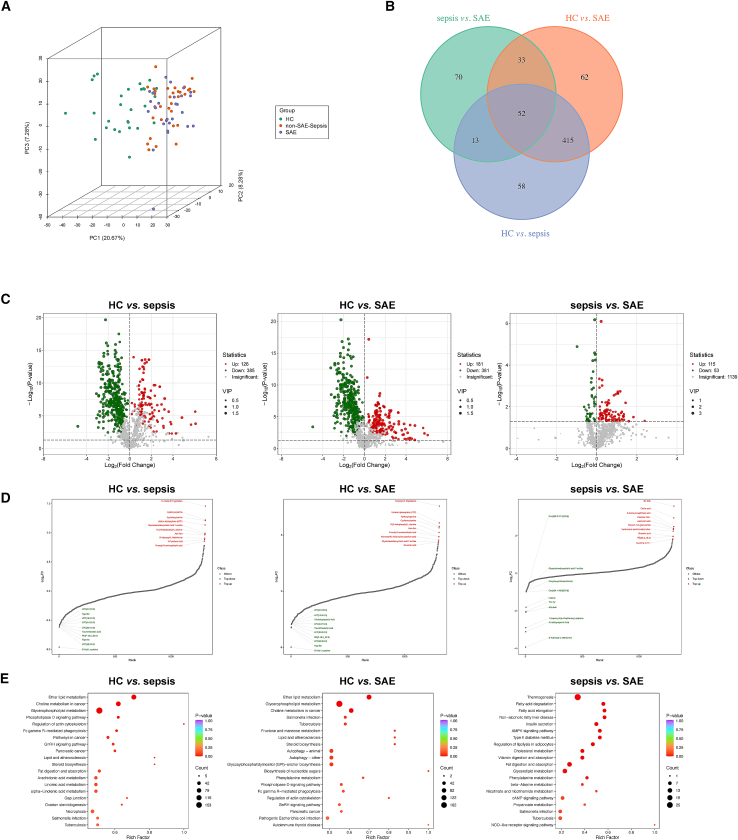
Metabolomic profile alterations and biological pathways in SAE (A) Three-dimensional PCA shows clear metabolic separation among HC, sepsis, and SAE groups. (B) Venn diagram of significantly altered metabolites (|log_2_FC| > 1, FDR <0.05) across pairwise comparisons. (C) Volcano plots comparing HC vs. sepsis, HC vs. SAE, and sepsis vs. SAE, highlighting significantly upregulated (red) and downregulated (green) metabolites defined by |log_2_FC| > 1 and FDR <0.05. VIP values (>1) were used only to rank metabolite importance in multivariate analyses and not as volcano plot thresholds. (D) Ranked importance of differential metabolites integrating VIP scores and fold changes. (E) KEGG pathway enrichment analysis for each comparison, with rich factor on the *x* axis and –log_10_(p) on the *y* axis; dot size denotes the number of metabolites and color intensity indicates enrichment significance.

To elucidate the metabolic differences between SAE, sepsis, and healthy states, we conducted multivariate and differential analyses on the preprocessed feature matrix. Three-dimensional principal component analysis (3D-PCA) revealed distinct clustering of samples from the HC group, sepsis group, and SAE group. The first three components captured the majority of the variance, with no significant overlap among the groups, supporting substantial metabolic disparities during the transition from health to sepsis and subsequently to brain dysfunction (Figure 2A).

Pairwise comparisons (HC vs. sepsis, HC vs. SAE, sepsis vs. SAE) using stringent criteria (|log_2_FC| > 1 and FDR-adjust ∗*p* < 0.05) identified 511, 562, and 168 significantly altered metabolites, respectively (Figure 2C). Venn diagram analysis showed that 52 metabolites were consistently dysregulated across all three comparisons, suggesting their involvement in the progression from systemic infection to brain dysfunction. Additionally, unique subsets of differential metabolites (e.g., 70 specifics to sepsis vs. SAE, 62 specifics to HC vs. SAE) suggested stage-specific or SAE-specific metabolic alterations (Figure 2B).

Differential metabolites were ranked based on their discriminatory importance by integrating VIP scores and absolute fold changes (Figure 2D). The top-ranked metabolites predominantly belonged to glycerophospholipids, phosphatidylcholines (and fatty acid derivatives), organic acids, as well as amino acids and their metabolites.

KEGG pathway enrichment analysis was subsequently performed to investigate pathway alterations across disease stages (Figure 2E). Compared to the HC group, the top three enriched pathways in both the sepsis and SAE groups were ether lipid metabolism, choline metabolism in cancer, and glycerophospholipid metabolism. All three pathways are closely associated with phospholipid metabolism, which constitutes a major structural component of cell membranes. Thus, dysregulation of cell membrane function may represent a key mechanism underlying sepsis-induced brain dysfunction.

In the sepsis vs. SAE comparison, the top three enriched pathways were thermogenesis, fatty acid degradation, and fatty acid elongation. These findings suggest that energy metabolism may be altered during the transition from sepsis to encephalopathy, with increased mobilization and degradation of fatty acids to sustain energy supply, accompanied by abnormal thermogenic activity. Previous studies have reported that abnormal fatty acid oxidation not only promotes reactive oxygen species (ROS) production, damaging neurons and glial cells, but its metabolites may also exhibit neurotoxicity.^11^ Consequently, this metabolic shift may exacerbate neuroinflammation and contribute to the development of encephalopathy.

### Development and validation of machine learning models for SAE discrimination

**Figure 3:**
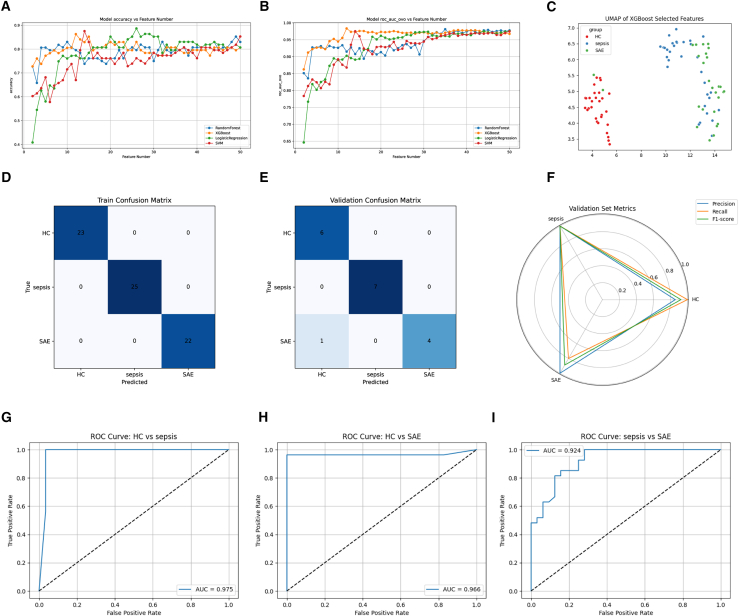
Performance of metabolomics-based classifiers for SAE identification (A and B) Comparative accuracy of four algorithms (RF, XGBoost, LR, and SVM) and average multiclass ROC AUC across feature set sizes. (C) UMAP projection of XGBoost-selected features showing clear separation among HC (red), sepsis (blue), and SAE (green). (D and E) Confusion matrices for the training set (*n* = 70) and independent validation set (*n* = 18). (F) Radar plot summarizing macro-precision, recall, and F1-score in the validation cohort. (G–I) ROC curves for binary classifications (HC vs. sepsis, HC vs. SAE, sepsis vs. SAE) with corresponding AUCs, confirming robust diagnostic performance.

To enhance discriminatory resolution among HC, sepsis, and SAE, we systematically evaluated four classifiers (RF, XGBoost, LR, and SVM) across varying feature set sizes (Figures 3A and 3B). XGBoost exhibited superior efficiency, achieving ≥0.90 classification accuracy and maintaining one-versus-one multiclass AUC >0.96 with only 12–15 selected metabolites, outperforming the alternative algorithms. UMAP visualization of the optimized feature subset demonstrated tight, well-separated clusters corresponding to HC, sepsis, and SAE, confirming the capacity of the selected metabolomic signature to resolve clinical phenotypes in low-dimensional space (Figure 3C). In the training data (*n* = 70), the XGBoost model achieved perfect classification across all three groups (Figure 3D). On the independent validation cohort (*n* = 18), only one SAE sample was misclassified as HC, yielding an overall accuracy of 0.93 (Figure 3E). Macro-averaged precision, recall, and F1 scores remained high (≥0.82), indicating consistent, balanced performance (Figure 3F). Binary ROC analyses further underscored robustness: the model discriminated HC vs. sepsis, HC vs. SAE, and sepsis vs. SAE with AUCs of 0.975, 0.966, and 0.924, respectively (Figures 3G–3I), with sensitivity and specificity metrics approaching optimal values—particularly in distinguishing HC from sepsis.

This metabolic signature-based XGBoost classifier enables not only multi-class identification of SAE but also early differentiation between septic patients likely to develop encephalopathy and those without central involvement, offering a quantitative basis for targeted monitoring and timely intervention.

### Expression dynamics, specificity, and clinical relevance of key metabolic drivers

**Figure 4:**
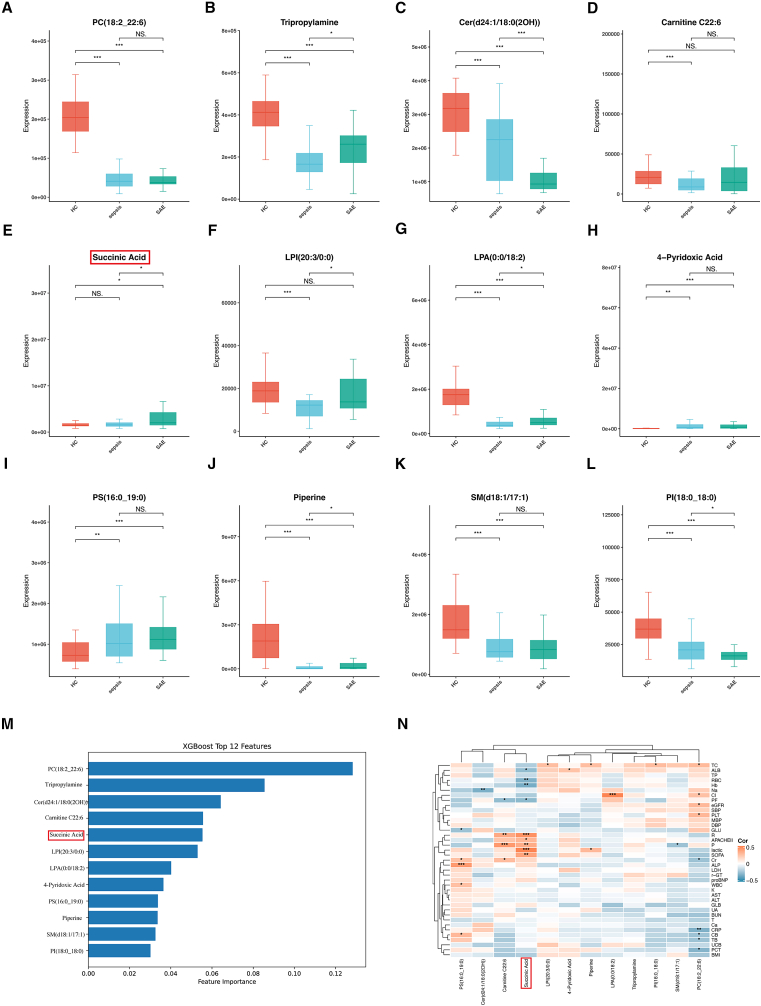
Expression dynamics, specificity, and clinical relevance of key metabolic drivers (A–L) Boxplots of the 12 highest-ranking XGBoost-selected metabolites across HC, sepsis, and SAE groups. (M) Feature importance ranking in the final XGBoost model. (N) Spearman correlation heatmap showing associations between these metabolites and clinical indices (APACHE II, SOFA, lactate, CRP, eGFR, albumin, etc.); red indicates positive correlation, blue indicates negative correlation, and asterisks denote significance. Data are presented as mean ± SD, ∗*p* < 0.05, ∗∗*p* < 0.01, ∗∗∗*p* < 0.001.

Interpretability analysis of the final constructed XGBoost model identified 12 metabolites with the greatest contribution to the outcome. These encompass membrane lipids (e.g., glycerophospholipids and sphingolipids), central carbon metabolism intermediates (succinate), amines (tripropylamine), vitamin breakdown products (4-pyridoxic acid), and exogenous signaling molecules (piperine) (Figure 4M).

During the progression from HC to sepsis and further deterioration to SAE, the metabolic profile demonstrates dynamic and stage-specific alterations. Overall, most membrane lipid components exhibited a consistent downward trend, including PC(18:2_22:6, Figure 4A), Cer(d24:1/18:0(2OH), Figure 4C), SM(d18:1/17:1, Figure 4K), and PI(18:0_18:0, Figure 4L). This decreasing pattern was highly significant in both the HC vs. sepsis and sepsis vs. SAE comparisons, indicating a progressive loss of key lipid structural elements and neuroprotective lipid signaling pathways during the development of encephalopathy. In sharp contrast to the general decline described previously, some metabolites displayed more complex dynamic trajectories. For example, components such as tripropylamine (Figure 4B), docosahexaenoyl-carnitine (Carnitine C22:6, Figure 4D), lyso-phosphatidylinositol LPI(20:3/0:0, Figure 4F), piperine (Figure 4J), and lysophosphatidic acid LPA(0:0/18:2, Figure 4G) showed higher levels in HC, decreased upon progression to sepsis, but rebounded during the development of SAE. This unique “decrease-first-increase-later” pattern suggests their potential involvement in specific pathophysiological processes at different stages of the disease. Among the 12 differentially expressed metabolites systematically screened, the concentration trend of succinate (Figure 4E) was particularly noteworthy, demonstrating a stepwise increase from HC to sepsis and further to SAE (∗∗*p* < 0.05). This strongly implies that succinate accumulation is closely associated with the initiation and worsening of brain dysfunction. In contrast, 4-pyridoxic acid (Figure 4H) and phosphatidylserine PS (16:0_19:0, Figure 4I) did not show a further increase in SAE compared to sepsis, suggesting that they may be primarily related to the early systemic inflammatory response in sepsis rather than directly driving the pathogenesis of SAE.

To further evaluate the importance of these metabolic changes, we performed a feature importance analysis. The results showed that the top five metabolites with the highest contributions were, in order: phosphatidylcholine PC(18:2_22:6), tripropylamine, ceramide Cer(d24:1/18:0(2OH)), carnitine C22:6, and succinate. Among these, widespread lipid depletion combined with the specific elevation of succinate levels collectively constitutes a characteristic metabolic disturbance that distinguishes SAE from both sepsis and HC (Figure 4M).

To investigate the association between differential metabolites and the prognosis of patients with SAE, we performed Spearman correlation analysis between the 12 differential metabolites and clinical patient data (Figure 4N). The results indicated that succinate was positively correlated with APACHE II score, SOFA score, and lactate level, suggesting that the accumulation of succinate may be a key mechanism driving clinical deterioration and could serve as a biomarker for poor prognosis in SAE. Other metabolites, such as PC(18:2_22:6), showed a positive correlation with estimated glomerular filtration rate and a negative correlation with creatinine, indicating that lipid metabolism may play an important role in the pathophysiology of sepsis-induced kidney injury.

### Succinate exacerbates cognitive dysfunction in CLP-induced SAE mice

**Figure 5:**
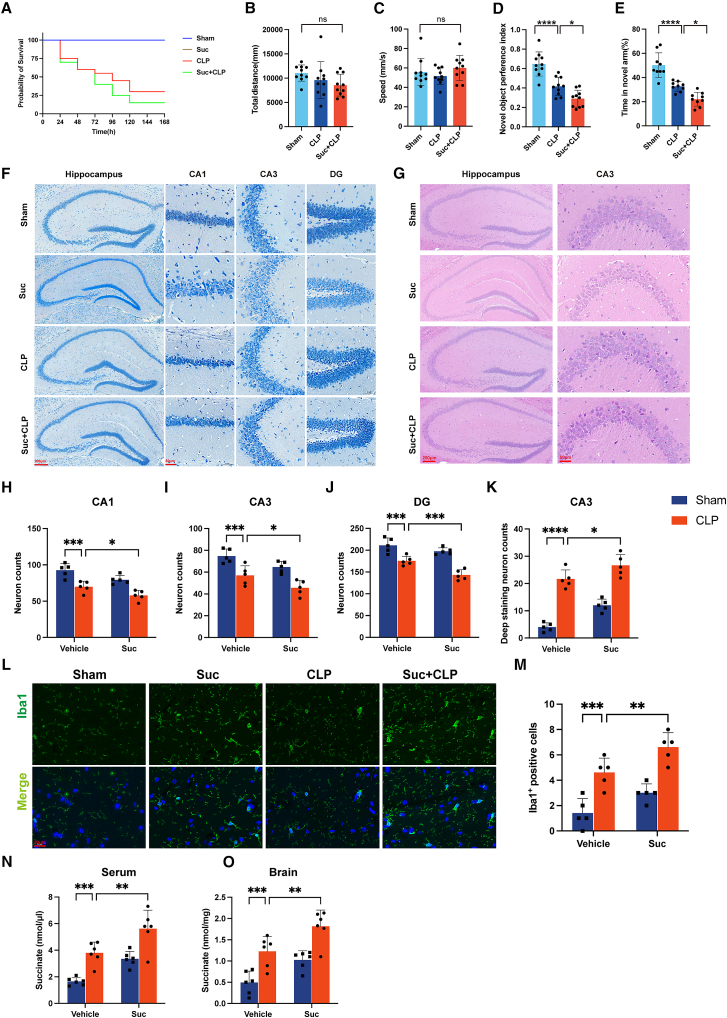
Succinate exacerbates cognitive dysfunction in CLP-induced SAE mice (A) Kaplan-Meier survival curves of mice in the sham, Suc, CLP, and Suc+CLP groups (*n* = 20/group). (B and C) Open-field test showing total distance traveled and average speed (*n* = 10/group). (D) Novel object recognition test and (E) Y-maze test assessing cognitive performance (*n* = 10/group). (F) Nissl staining of hippocampal subregions (CA1, CA3, DG) (*n* = 5/group). Scale bars: 200 μm (overview), 50 μm (enlarged view). (G) Hematoxylin-eosin (HE) staining of hippocampal sections (*n* = 5/group). Scale bars: 200 μm (overview), 50 μm (enlarged view). (H–J) Quantification of Nissl-stained neurons in hippocampal CA1, CA3, and DG regions (*n* = 5/group). (K) Quantification of deeply stained degenerative neurons in hippocampal CA3 regions (*n* = 5/group). (L) Immunofluorescence staining showing microglial activation in hippocampal sections (*n* = 5/group). Scale bar: 50 μm. (M) Quantification of Iba1^+^ microglial cells in hippocampal sections (*n* = 5/group). Scale bar: 20 μm. (N and O) Succinate concentrations measured in (N) serum and (O) brain tissue (*n* = 6/group). Data are presented as mean ± SD, one-way ANOVA followed by Tukey’s post hoc test. ∗*p* < 0.05, ∗∗*p* < 0.01, ∗∗∗*p* < 0.001, ∗∗∗∗*p* < 0.0001.

To clarify the role of succinate in SAE, mice were administered 1.5% (wt/vol) succinate or vehicle in drinking water for three weeks before CLP. Succinate supplementation significantly increased mortality in septic mice (Figure 5A). In the open-field test, total distance traveled and average speed did not differ among groups (Figures 5B and 5C), excluding locomotor impairment as a confounder. However, cognitive function was markedly compromised in the Suc + CLP group, as indicated by a lower novel object preference index and reduced time spent in the novel arm of the Y-maze compared with the CLP group (Figures 5D and 5E). Histopathological analysis revealed pronounced neuronal damage in the hippocampus of succinate-treated mice, characterized by cellular disorganization and reduced Nissl body density (Figures 5F and 5G). Quantification confirmed significant neuronal loss in the CA1, CA3, and DG regions, as well as increased numbers of degenerative neurons (Figures 5H–5K).

Immunofluorescence staining further showed enhanced microglial activation in the hippocampus, evidenced by a higher density of Iba1^+^ activated microglia (Figures 5L and 5M). To verify systemic succinate exposure, we measured succinate concentrations in both serum and brain tissues. Compared with the sham group, the succinate supplementation group exhibited significantly elevated succinate levels, confirming effective absorption from oral administration (Figures 5N and 5O).

These findings confirm that the CLP model, with or without exogenous supplementation, reproduces the metabolic dysregulation of succinate observed in SAE patients, thereby validating its translational relevance. To further examine the pro-inflammatory effects of succinate, BV2 microglial cells were treated with exogenous succinate *in vitro*. qPCR analysis revealed significant upregulation of IL-1β, TNF-α, and IL-6 expression compared with controls, indicating that succinate directly enhances microglial inflammatory activation (Figure S1).

## Discussion

SAE is an acute, diffuse brain dysfunction secondary to sepsis that must be differentiated from central nervous system infections or other metabolic encephalopathies.^12^ Elucidating the pathophysiological features of SAE is crucial for early identification, diagnosis, and treatment, which ultimately help reduce its high morbidity and mortality. In this study, by integrating widely targeted metabolomics with machine learning, we identified a distinct metabolic profile in SAE characterized primarily by succinate accumulation and disrupted glycerophospholipid metabolism.

Succinate is a key metabolic intermediate in the tricarboxylic acid (TCA) cycle, converted from the precursor succinyl-CoA by succinyl-CoA synthetase with concomitant production of GTP or ATP.^13^ Emerging evidence indicates that succinate is not merely a passive metabolite: its abnormal accumulation exerts pro-inflammatory and detrimental effects across diseases.^14^^,^^15^ Elevated succinate has been associated with mitochondrial dysfunction, apoptosis, and NLRP3 inflammasome activation,^16^^,^^17^^,^^18^ as well as chronic inflammatory disorders, such as inflammatory bowel disease, atherosclerosis, and rheumatoid arthritis.^19^^,^^20^

In our study, metabolomic profiling of HC, sepsis, and SAE groups revealed a marked elevation of succinate in SAE, which correlated with disease severity,^21^ implicating succinate as a potential metabolic driver of SAE progression. Previous studies have shown that inhibition of succinate oxidation alleviates ischemic brain injury, whereas exogenous succinate worsens neuronal damage and impairs neural stem-cell proliferation.^22^^,^^23^ Moreover, succinate accumulation has been linked to immune dysregulation, including FOXP3 destabilization and Treg dysfunction in inflammatory bowel disease.^24^ Likewise, the detrimental effects of succinate have been observed in lung tissue. Mechanistically, in LPS-stimulated macrophages the TCA cycle is remodeled, and accumulated succinate drives ROS and IL-1β production through SDH, amplifying inflammation.^25^ Although direct measurements of SDH activity or ROS were not performed in brain tissue, our *in vitro* results in BV2 microglia demonstrated that exogenous succinate markedly enhanced the transcription of IL-1β, TNF-α, and IL-6. Similarly, in other organs such as the lung, elevated succinate promotes M1 macrophage polarization and epithelial apoptosis, further underscoring its pro-inflammatory potential.^26^ Consistently, in our CLP-induced SAE model, exogenous succinate supplementation aggravated neuroinflammation and cognitive dysfunction. Collectively, these findings position succinate as a central mediator of inflammatory injury, suggesting that targeting succinate metabolism or signaling may represent a potential therapeutic strategy for SAE.

The pronounced elevation of succinate observed in SAE likely reflects metabolic reprogramming under septic stress. First, mitochondrial dysfunction may impair electron transport and inhibit or reverse SDH flux, leading to intracyclic accumulation of succinate within the TCA cycle.^27^ Second, a shift toward aerobic glycolysis, driven by inflammatory and hypoxic signaling through HIF-1α, enhances anaplerotic input from substrates such as glutamine and GABA, thereby increasing succinate generation.^28^ These intertwined processes, mitochondrial impairment and glycolytic activation, may jointly account for the marked rise in systemic and cerebral succinate seen in both patients and experimental SAE, consistent with findings from ischemia-related studies where limiting succinate oxidation mitigated tissue injury.^23^ Together, these data suggest that succinate accumulation represents both a marker and a metabolic amplifier of neuroinflammation, bridging mitochondrial dysfunction with immune activation in SAE. Within the context of sepsis, our findings further support a model in which systemic infection and inflammation first render the brain metabolically vulnerable, and elevated succinate then amplifies microglial activation and neuronal injury on this background. In this way, succinate acts as a metabolic amplifier of sepsis-induced encephalopathy rather than as an isolated neurotoxin.

In addition to succinate, this study identified significant disruptions in glycerophospholipid metabolism in SAE patients. Glycerophospholipids are the most abundant class of phospholipids in cell membranes, forming the core framework of the phospholipid bilayer and participating in various physiological processes, such as signal transduction, maintenance of membrane stability, membrane trafficking, and energy storage.^29^ In the central nervous system, glycerophospholipids are particularly important as major components of neuronal membranes and myelin.^30^ In this study, machine learning analysis of serum metabolomics from HC, sepsis patients, and SAE patients revealed significant differences in glycerophospholipid metabolism. This finding is consistent with previous studies reporting that disruptions in glycerophospholipid metabolism are associated with the onset of depression in animal models.^31^^,^^32^ Similarly, a study using high-resolution demultiplexed ion mobility spectrometry (HRdm-IMS) technology identified abnormal glycerophospholipid metabolism in the hippocampus and cortex of a mouse model of Alzheimer’s disease.^33^ Combined with our results, aberrant glycerophospholipid metabolism may represent an early pathological process in SAE. This metabolic disturbance may contribute to the development and progression of SAE by affecting neuronal membrane structure, myelin function, and neural signal transmission. Several metabolites displayed a “decrease-first-increase-later” trend across the progression of SAE. This dynamic fluctuation may reflect early consumption of energy substrates and structural lipids during the acute phase of systemic inflammation, leading to transient depletion of critical metabolites.^34^ As the inflammatory response stabilizes and mitochondrial function partially recovers, these metabolites may rebound due to compensatory metabolic reprogramming.^35^ Lipid remodeling under oxidative stress and microglial activation may further drive this compensatory increase.

### Conclusions

In summary, by integrating metabolomics with machine learning, we identified succinate accumulation and impaired glycerophospholipid metabolism as major contributors to the pathophysiology of SAE. These findings not only provide potential biomarkers for early diagnosis of SAE but also suggest that targeting the succinate pathway and glycerophospholipid metabolism may offer novel therapeutic strategies to improve the prognosis of SAE.

### Limitations of the study

This study has several limitations. First, although exogenous succinate supplementation provided gain-of-function evidence that elevated succinate aggravates neuroinflammatory injury, loss-of-function validation was not performed. The receptor-mediated mechanisms, therefore, remain to be elucidated. Future work will incorporate pharmacologic inhibition of SUCNR1, SUCNR1 knockdown in microglia, and SUCNR1-deficient mouse models to establish causality. If SUCNR1 antagonism or deficiency were found to attenuate neuroinflammation and cognitive deficits in this model, it would strongly support receptor-mediated signalling—rather than nonspecific metabolic toxicity—as a primary mechanism and would highlight SUCNR1 as a potential therapeutic target in SAE. Second, our chronic succinate supplementation protocol does not fully replicate the hyperacute onset of SAE. However, it was deliberately chosen to model sustained metabolic stress during and after sepsis, which is likely to contribute to delayed neuronal injury and long-term cognitive impairment. In this sense, the model represents both a limitation and a strength, as it captures a clinically relevant, metabolically stressed SAE phenotype rather than only the immediate-early phase. Third, while 12 differential metabolites were identified, their specific biological functions and dynamic changes during disease progression were not investigated. Fourth, complementary validation using quantitative targeted assays and multi-omics integration (proteomics, transcriptomics) will be needed to construct causal regulatory networks. Fifth, despite blood sampling preceding encephalopathy onset, the retrospective nested case-control design primarily demonstrates association rather than prediction; prospective cohort studies are required to confirm predictive utility and clinical applicability. Finally, potential biases due to sample size and data variability should be addressed in future studies to enhance the robustness and generalizability of our findings. In addition, while sex and gender may influence both metabolic profiles and neuroinflammatory responses, our study did not account for their potential effects. Future studies should consider these variables to determine their impact on the outcomes observed and to provide a more comprehensive understanding of the mechanisms involved in SAE.

## Resource availability

### Lead contact

Further information and requests for resources and reagents should be directed to and will be fulfilled by the lead contact, Wei Zhu (tjjzkzw512@tjh.tjmu.edu.cn).

### Materials availability

This study did not generate new unique reagents.

### Data and code availability

• Data: Data reported in this paper will be shared by the lead contact upon request.
• Code: This paper does not report original code.
• Other: Any additional information required to reanalyze the data reported in this paper is available from the lead contact upon request.

## Acknowledgments

This research was supported by Key R&D Program Projects in Hubei Province (Reference: 2022BCA038).

## Author contributions

Conceptualization, methodology, W.Z. and Y.S.; investigation, validation, writing – original draft preparation and visualization, H.H. and Y.F.; review & editing, S.W. and S.P.; writing – review & editing, Y.L.; data curation, D.L., J.C., and Y.Z. All authors agree to be accountable for all aspects of work ensuring integrity and accuracy.

## Declaration of interests

The authors declare no competing interests.

## STAR★Methods

### Key resources table

**Table:** 

REAGENT or RESOURCE	SOURCE	IDENTIFIER
**Antibodies**		

Anti-IBA1 Guinea pig antibody	Synaptic Systems	Cat#234308; RRID: AB_2924932

**Biological samples**		

Human plasma	Tongji Hospital of Tongji Medical College of Huazhong University of Science and Technology	N/A

**Critical commercial assays**		

RevertAid Reverse Transcriptase	Thermo	Cat#EP0442
SYBR Green Master	Roche	Cat#4913914001
TRIzol reagents	Invitrogen	Cat#15596018
Succinate Assay Kit	Abcam	Cat#ab204718

**Experimental models: Cell lines**		

Mouse: BV2	Pricella	Cat NO.: CL-0493

**Experimental models: Organisms/strains**		

Mouse:C57BL/6J	Hubei Biont Biological Technology Co., Ltd	N/A

**Oligonucleotides**		

Mouse IL-1β qPCR primers	F:GTGTCTTTCCCGTGGACCTT R:TCATCTCGGAGCCTGTAGTG	N/A
Mouse IL-6 qPCR primers	F:TACCACTTCACAAGTCGGAG R:CTGCAAGTGCATCATCGTTG	N/A
Mouse TNF-α qPCR primers	F:GTGTCTTTCCCGTGGACCTT R:GCCATAGAACTGATGAGAGG	N/A
Mouse β-actin qPCR primers	F:CATTGCTGACAGGATGCAGA R:TGCTGGAAGGTGGACAGTGA	N/A

**Software and algorithms**		

SlideViewer2.6	3DHISTECH	https://www.3dhistech.com/
GraphPad Prism 8	Graphpad	https://www.graphpad.com/
Python 3.8	Python	https://www.python.org/
ImageJ	NIH	https://imagej.net/software/imagej/
Analyst 1.6.3	Sciex	https://sciex.com/

### Experimental model and study participant details

#### Study population and ethical considerations

This study was conducted as a retrospective nested case–control design.  A total of 88 participants were enrolled, including 29 healthy controls (HC), 32 patients with sepsis without encephalopathy, and 27 patients with SAE. Septic patients admitted to the intensive care unit (ICU) of Tongji Hospital between March 2023 and July 2024 were screened. Clinical data were collected from electronic data capture systems and case report forms. Sepsis was diagnosed based on an acute increase in the total Sequential Organ Failure Assessment (SOFA) score of ≥2 points resulting from infection.^36^ Diagnoses of both sepsis and SAE were independently confirmed by two experienced ICU physicians.

Healthy control (HC) subjects, who had a SOFA score of 0 and no evidence of infection, were recruited from a health examination center to serve as the control group.

The study was registered with the Chinese Clinical Trial Registry (Registration No.: chictr220064100) and approved by the Medical Ethics Committee of Tongji Hospital, Tongji Medical College, Huazhong University of Science and Technology (IRB: TJ-IRB20220767). Written informed consent was obtained from all participants or their legally authorized representatives.

#### Animal experiments

According to previous studies, the SAE model was established using cecal ligation and puncture (CLP).^37^ Briefly, C57BL/6J male mice were provided with drinking water containing 1.5% (wt/vol) sodium succinate or regular water (control) for 3 weeks, and the water was refreshed every other day.^38^^,^^39^ After this pre-treatment, mice were subjected to CLP surgery to induce sepsis. Postoperative survival was monitored, and animals were sacrificed at designated time points for sample collection. Animals were randomly assigned to experimental groups using a random-number table, and all behavioral, histological, and biochemical assessments were performed by investigators blinded to group allocation. All experimental protocols were approved by the Animal Ethics Committee of Tongji Hospital, Tongji Medical College, Huazhong University of Science and Technology (approval no. TJH-202405011) and were conducted in accordance with the ARRIVE 2.0 guidelines and the National Institutes of Health Guide for the Care and Use of Laboratory Animals.

#### Cell culture

BV2 murine microglial cells were cultured in high-glucose DMEM supplemented with 10% fetal bovine serum and 1% penicillin–streptomycin at 37°C in a humidified 5% CO_2_ incubator. Cells were passaged at 70–80% confluence and seeded at the desired density before treatment with succinate and/or LPS as indicated in the experiments. All cells used in this study were verified to be free from mycoplasma contamination by PCR and/or Immunofluorescence.

### Method details

#### Sample preparation

Blood samples were collected within 24 hours after patients met the diagnostic criteria for sepsis. The diagnosis of SAE was subsequently established during hospitalization, based on neurological assessment and exclusion of alternative causes. Accordingly, patients were stratified into sepsis without encephalopathy and SAE groups for further analysis. Blood specimens from both patients and HC are temporarily stored in serum separator tubes. After standing at room temperature for 60 minutes, the samples are centrifuged for 10 minutes in a refrigerated centrifuge (1600 g, 4°C). The supernatant is then aliquoted and stored at -80°C for subsequent liquid chromatography-tandem mass spectrometry (LC-MS/MS) analysis.

#### Extraction of hydrophobic compounds

The sample was taken out from the -80°C refrigerator, thawed on ice and vortexed for 10 s. Mix 50 μL of the sample and 1mL of the extraction solvent (Methyl tert-butyl ether (MTBE): MeOH =3:1, v/v) containing internal standard mixture. After whirling the mixture for 15 min, 200 μL of ultrapure water was added. Vortex for 1 min and centrifuge at 12,000 rpm for 10 min. 200 μL of the upper organic layer was collected and evaporated using a vacuum concentrator. The dry extract was dissolved in 200 μL reconstituted solution (ACN: Isopropanol (IPA)=1:1, v/v) to LC-MS/MS analysis.

#### Ultra performance liquid chromatography (UPLC) conditions

The sample extracts were analyzed using an LC-ESI-MS/MS system (UPLC, ExionLC AD, https://sciex.com.cn/; MS, QTRAP®6500+ System, https://sciex.com/).

#### UPLC conditions for hydrophilic compounds

The analytic0formic acid, 10 mmol/L ammonium formate), B: acetonitrile/isopropanol (10/90 V/V, 0.1% formic acid, 10 mmol/L ammonium formate); gradient program, A/B (80:20, V/V) at 0 min, 70:30 V/V at 2.0 min, 40:60 V/V at 4 min, 15:85 V/V at 9 min, 10:90 V/V at 14 min, 5:95 V/V at 15.5 min, 5:95 V/V at 17.3 min, 80:20 V/V at 17.3 min, 80:20 V/V at 20 min; flow rate, 0.35 ml/min; temperature, 45 °C; Injection volume: 2μl. The effluent was alternatively connected to an ESI-triple quadrupole-linear ion trap (QTRAP)-MS.

#### ESI-Q TRAP-MS/MS

LIT and triple quadrupole (QQQ) scans were acquired on a triple quadrupole-linear ion trap mass spectrometer (QTRAP), QTRAP® LC-MS/MS System, equipped with an ESI Turbo Ion-Spray interface, operating in positive and negative ion mode and controlled by Analyst 1.6.3 software (Sciex).

#### ESI-Q TRAP-MS/MS for hydrophilic compounds

The ESI source operation parameters were as follows: source temperature 500 °C; ion spray voltage (IS) 5500 V (Positive), -4500 V (Negative); ion source gas 1 (GS1), gas 2 (GS2), and curtain gas (CUR) were set at 55, 60, and 25.0 psi, respectively; the collision gas (collision-activated dissociation, CAD) was set high. Instrument tuning and mass calibration were performed with 10 and 100 μmol/L polypropylene glycol solutions in QQQ and LIT modes, respectively. A specific set of multiple reaction monitoring (MRM) transitions were monitored for each period according to the metabolites eluted within this period.

#### ESI-Q TRAP-MS/MS for hydrophobic compounds

The ESI source operation parameters were as follows: ion source, turbo spray; source temperature 500 °C; ion spray voltage (IS) 5500 V(Positive), -4500 V(Negative); ion source gas 1 (GS1), gas 2 (GS2), and curtain gas (CUR) were set at 45, 55, and 35 psi, respectively; the collision gas (CAD) was set medium. Instrument tuning and mass calibration were performed with 10 and 100 μmol/L polypropylene glycol solutions in QQQ and LIT modes, respectively. QQQ scans were acquired as MRM experiments with collision gas (nitrogen) set to 5 psi. Declustering potential (DP) and collision energy (CE) for individual MRM transitions was done with further DP and CE optimization. A specific set of MRM transitions were monitored for each period according to the metabolites eluted within this period.

#### Machine learning analysis

Raw LC–MS/MS data were preprocessed by peak extraction, retention-time alignment, deconvolution, and normalization, yielding 1,307 QC-vetted, non-redundant metabolite features; features were classified as “QC-vetted” if they met predefined thresholds for signal intensity, retention-time stability, and relative standard deviation in pooled quality-control samples. For data exploration, we applied unsupervised methods (PCA and UMAP) to visualize global structure and clustering.

To prevent data leakage, the full cohort (n = 88) was first split by stratified sampling into a training/validation set (n = 70) and an independent test set (n = 18) before any feature selection, preprocessing, or model fitting; a fixed random seed was used for reproducibility. All model development steps, feature ranking and selection, preprocessing, hyperparameter tuning, and cross-validation performance estimation, were performed exclusively within the training/validation set. The independent test set remained untouched and was used only once for final evaluation.

Supervised modeling followed a multi-class feature-selection and classification pipeline. Candidate features were ranked by recursive feature elimination (RFE) informed by variable-importance measures from random forest (RF) and support vector machine (SVM) models. Feature selection was implemented inside each training fold to avoid leakage and to allow assessment of selection stability across folds. We evaluated four classifiers: logistic regression (LR), RF, SVM, and extreme gradient boosting (XGBoost).

Preprocessing was model-specific and implemented within fold pipelines: standardization was applied for LR and SVM; no scaling was applied for tree-based methods. Hyperparameters were tuned using grid search nested within the 5-fold stratified cross-validation loop so that tuning and performance estimation remained strictly separated. For XGBoost we searched empirically constrained ranges. The primary selection metric during tuning was multiclass AUROC (one-vs-rest, macro-averaged); secondary metrics included accuracy, precision, recall, and F1-score. Where appropriate, ties in AUROC were resolved by preferring models with lower validation log-loss and greater feature-selection stability.

After hyperparameter selection, the final model was retrained on the entire training/validation set and evaluated once on the independent test set to estimate generalization performance. Test-set metrics (AUROC, accuracy, precision, recall, F1 and confusion matrix) are reported in the Results.

#### Nissl staining

Paraffin-embedded, paraformaldehyde-fixed brains were cut into 4 μm coronal hippocampal sections, deparaffinized, and rehydrated. Sections were stained with Nissl solution, briefly differentiated in ethanol, dehydrated, cleared in xylene, and mounted with neutral resin. Nissl-positive neurons in CA1, CA3 and DG regions were quantified under a microscope.

#### Hematoxylin and eosin (HE) staining

Paraffin sections of the hippocampus were deparaffinized, rehydrated, and stained with hematoxylin followed by eosin using standard procedures. After dehydration and clearing, sections were mounted and hippocampal structure and neuronal injury were assessed by microscopy.

#### Immunofluorescence staining

Hippocampal paraffin sections were deparaffinized and subjected to heat-mediated antigen retrieval in citrate buffer, then blocked with normal serum. Sections were incubated with Guinea pig anti-Iba1 primary antibody overnight at 4°C, followed by fluorophore-conjugated secondary antibody and DAPI nuclear counterstaining. Slides were mounted with antifade medium, and Iba1-positive microglia in the hippocampus were imaged and quantified by fluorescence microscopy.

#### Succinate quantification

Succinate concentrations in plasma and brain were measured using the Abcam Succinate Assay Kit^40^ (ab204718) according to the manufacturer’s instructions. Briefly, plasma aliquots (10 μL) were diluted 1:5 and brain tissue (∼10 mg) was homogenized in 100 μL extraction buffer, with 10 μL of supernatant diluted 1:5 for assay. Measurements were converted to absolute concentrations using the kit standard curve, and results were adjusted for dilution factors.

### Quantification and statistical analysis

All quantitative data are expressed as mean ± standard deviation (SD). Statistical analyses were performed using GraphPad Prism 8.0. Group comparisons were evaluated by two-tailed Student’s t test or one-way analysis of variance (ANOVA) followed by Tukey’s post hoc test for multiple comparisons. Data normality was assessed using the Shapiro–Wilk test. Survival curves were analyzed using the Kaplan–Meier method and compared with the log-rank test. Correlations between variables were examined using Spearman’s rank correlation coefficient for non-parametric data. All tests were two-sided, and a P value < 0.05 was considered statistically significant. Each experiment was independently repeated at least twice to ensure reproducibility.

### Additional resources

The study was registered with the Chinese Clinical Trial Registry (Registration No.: chictr220064100) and approved by the Medical Ethics Committee of Tongji Hospital, Tongji Medical College, Huazhong University of Science and Technology (IRB: TJ-IRB20220767). Written informed consent was obtained from all participants or their legally authorized representatives. More information can be found on the Chinese Clinical Trial Registry(https://www.chictr.org.cn/showproj.html?proj=174763).
