# Supplementary material for: Widely targeted metabolomics and machine learning identify succinate as a key metabolite in sepsis-associated encephalopathy
Source: iScience. 2025 Dec 23;29(1):114520. doi: 10.1016/j.isci.2025.114520 (PMC12809278; doi:10.1016/j.isci.2025.114520)
Supplement: Document S1. Figure S1 [file mmc1.pdf]

iScience, Volume *29*

Supplemental information

Widely targeted metabolomics and machine learning identify succinate as a key metabolite in sepsis-associated encephalopathy

Hongjie Hu, Yikuan Feng, Yunxi Zhou, Shu Peng, Dayong Li, Shuhui Wu, Hebin Jiang, Yuru Lu, Jingbo Chen, Yaqin Song, and Wei Zhu

Widely Targeted Metabolomics and Machine Learning Identify Succinate as a Key Metabolite in Sepsis-Associated Encephalopathy

Hongjie Hu[1,2†], Yikuan Feng[1,2†], Yunxi Zhou[1,2], Shu Peng[3], Dayong Li[1,2], Shuhui Wu[1,2], Hebin Jiang[1,2], Yuru Lu[1,2], Jingbo Chen, Yaqin Song[1,2*], Wei Zhu[1,2*]

1. Department of Emergency Medicine, Tongji Hospital, Tongji Medical College, Huazhong University of Science and Technology, Wuhan, Hubei 430030, PR China;

2. Department of Intensive Care Medicine, Tongji Hospital, Tongji Medical College, Huazhong University of Science and Technology, Wuhan, Hubei 430030, PR China;

3. Department of Thoracic Surgery, Tongji Hospital, Tongji Medical College, Huazhong University of Science and Technology, Wuhan, Hubei 430030, PR China

* Correspondence:

Wei Zhu, MD, PhD. Department of Emergency Medicine, Department of Intensive Care Medicine, Tongji Hospital, Tongji Medical College, Huazhong University of Science and Technology, 1095 Jiefang Ave, Wuhan, Hubei, PR China, 430030 (Email: tjjzkzw512@tjh.tjmu.edu.cn)

Yaqin Song, MD, PhD. Department of Emergency Medicine, Department of Intensive Care Medicine, Tongji Hospital, Tongji Medical College, Huazhong University of Science and Technology, 1095 Jiefang Ave, Wuhan, Hubei, PR China, 430030 (Email: 18772954675@163.com)

[†] These authors have contributed equally to this work.

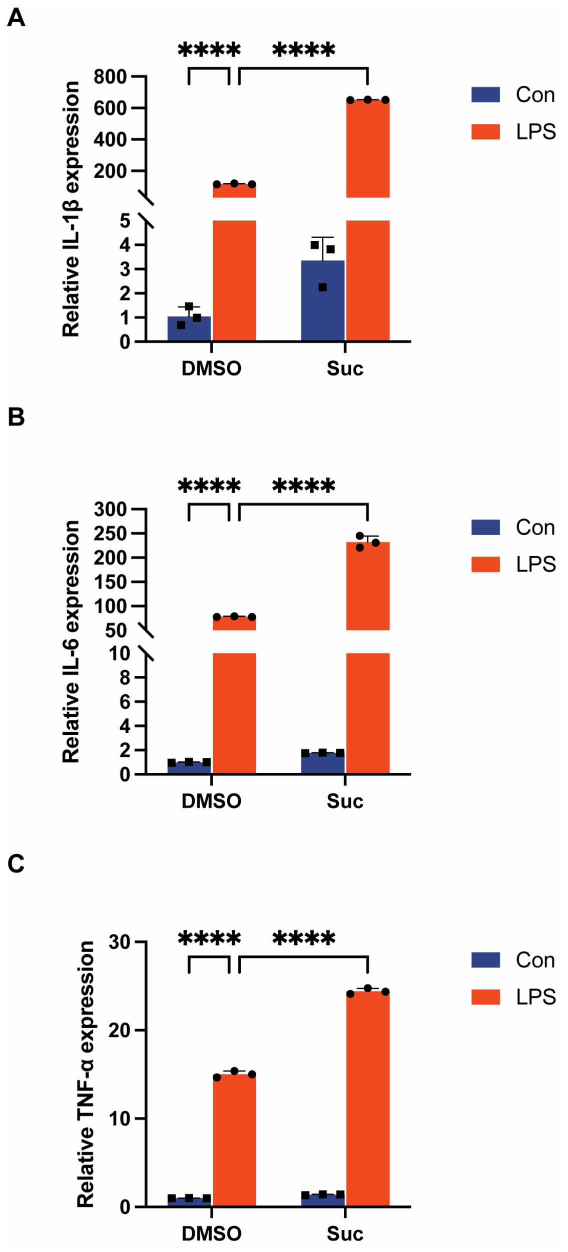

Supplementary Figure 1. Succinate enhances inflammatory cytokine expression in BV2 microglial cells.

(A–C) Relative mRNA expression levels of IL-1β (A), IL-6 (B), and TNF-α (C) in BV2 microglial cells after 24 h treatment with vehicle (DMSO) or succinate (Suc, 5 mM), with or without LPS stimulation (1000 ng/mL). Data are presented as mean ± SD (n = 3 independent experiments).****$p < 0.0001$, one-way ANOVA followed by Tukey's post hoc test.
